# Assessment of the Stress Response in North American Deermice: Laboratory and Field Validation of Two Enzyme Immunoassays for Fecal Corticosterone Metabolites

**DOI:** 10.3390/ani10071120

**Published:** 2020-06-30

**Authors:** Andreas Eleftheriou, Rupert Palme, Rudy Boonstra

**Affiliations:** 1 Wildlife Biology Program, University of Montana, 32 Campus Drive, FOR 109, Missoula, MT 59812, USA; 2 Department of Biomedical Sciences, University of Veterinary Medicine, A-1210 Vienna, Austria; rupert.palme@vetmeduni.ac.at; 3 Centre for the Neurobiology of Stress, University of Toronto Scarborough, 1265 Military Trail, Toronto, ON M1C 1A4, Canada; boonstra@utsc.utoronto.ca

**Keywords:** captivity-induced stress, enzyme immunoassay, fecal glucocorticoid metabolites, physiological stress in rodents

## Abstract

**Simple Summary:**

If we want to employ stress physiology in the management and conservation of wildlife populations, we need robust methods to quantify stress physiology in the field. Although this is typically done with blood glucocorticoids (GCs), scientists now increasingly use fecal cortisol/corticosterone metabolites (FCMs), which are metabolized GCs excreted in feces. However, immunoassays to measure FCMs need to be validated for each species. North American deermice (*Peromyscus maniculatus*; hereafter deermice) are commonly used in laboratory and field studies. Although a corticosterone radioimmunoassay (RIA) has been validated for deermice, there are no validated enzyme immunoassays (EIAs), which do not require radioactive materials. Through laboratory and field experiments, we validated two EIAs for measuring FCMs in deermice. Researchers can now use these EIAs to evaluate stress physiology in deermice without the need for radioactive materials.

**Abstract:**

Stress physiology is commonly employed in studies of wildlife ecology and conservation. Accordingly, we need robust and suitable methods to measure stress physiology in the field. Fecal cortisol/corticosterone metabolites (FCMs) are now increasingly being used to non-invasively evaluate adrenocortical activity; a measure of stress physiology. However, immunoassays that measure FCMs must be appropriately validated prior to their use and factors that can influence FCMs, such as trap-induced stress, must be considered. Deermice (*Peromyscus maniculatus*) are widely used in scientific studies so that developing methods that appropriately measure their adrenocortical activity is critical. In the laboratory, we tested the suitability of two enzyme immunoassays (EIAs; a corticosterone EIA, and a group-specific 5α-pregnane-3β,11β,21-triol-20-one EIA) in deermice by challenging individuals with dexamethasone and adrenocorticotropic hormone (ACTH). We found that dexamethasone suppressed FCM levels within ~10 h post injection whereas ACTH increased FCM levels within ~2 h post injection. In the field, we found that FCM levels generally increased with more time in trap confinement when using both EIAs. Although we acknowledge low sample sizes (N = 4), our results validated the two EIAs for use with FCMs from deermice.

## 1. Introduction

In recent decades, researchers have started to employ stress physiology more often as a tool to evaluate how natural and anthropogenic stressors can affect survival and reproductive success of wildlife populations. Given the widespread use of stress physiology in managing and conserving wildlife, identifying suitable and robust methods for evaluating stress physiology in every species is of paramount importance. This is critical because anthropogenic stressors can induce chronic stress in wildlife, which can lead to pathological perturbations [1,2].

Adrenocortical activity, a measure of stress physiology, is typically evaluated via blood glucocorticoids (GCs) and more recently via fecal cortisol/corticosterone metabolites (FCMs), which are metabolized GCs excreted in feces [2,3,4]. Evaluation of stress physiology via FCMs is non-invasive and avoids the acute stress effects of capture, handling, and venipuncture [1]. Although researchers are increasingly using FCMs, there are concerns about the methodology used to measure them, such as lack of validation [4]. Immunoassays are validated when they can detect expected changes in FCM levels [4]. Without validation, inference becomes less robust. Thus, immunoassays need to undergo analytical, physiological, and biological validations before they are used to measure FCMs in field settings [4]. Analytical validation may include intra- and inter-assay coefficients of variation and parallelism tests [4]. Validations also must be performed in every species because suitable immunoassays for measuring FCMs can vary even between closely related species [4,5,6].

North American deermice (*Peromyscus maniculatus*, hereafter deermice) are widely used in biomedical (e.g., [7]), physiological (e.g., [8]), and ecological (e.g., [9]) research. Hence, it may not be surprising that FCM evaluation in deermice has already been investigated by others where a corticosterone radioimmunoassay (RIA; [10,11]) and two corticosterone enzyme immunoassays (EIAs; [12,13]) have been used. Although the RIA was validated before use, the EIAs were not, which makes their use questionable [4]. In addition, all assays used antibodies that bind to corticosterone, which is the predominant GC in *Peromyscus* (e.g., [14]). However, intact corticosterone is essentially absent from feces so their use may be suboptimal [4]. For example, an EIA that used an antibody, which detects FCMs with a 5α-3β,11β-diol structure, demonstrated improved FCM detection in house mice (*Mus musculus*) compared with corticosterone EIAs [15]. Nevertheless, corticosterone immunoassays may still detect FCMs, albeit to a lesser degree, because of cross-reactivity between the corticosterone antibody and FCMs [4]. Commercial corticosterone immunoassays are also relatively easy to acquire and use, although expensive [4]. However, commercial RIAs, unlike EIAs, may be less appealing because they use radioactive materials and require a licensed laboratory for their use [4,16]. To the best of our knowledge, there have been no studies that compared or validated EIAs for measuring FCMs in deermice. 

We can physiologically validate immunoassays by using adrenocorticotropic hormone (ACTH) and dexamethasone (a synthetic steroid), which increase and decrease endogenous GC production, respectively [4]. However, to biologically validate immunoassays, we need to use stressors that are biologically relevant to the species of interest. Although immunoassays can be validated biologically if they can track diurnal rhythm changes in FCMs, this should be done in addition to other biological validations [4], such as live trapping, which can increase FCMs (e.g., [17,18]). Knowing when these trap-induced rises in FCMs manifest in feces is also of practical use because they can artificially increase FCMs and lead to erroneous results about baseline adrenocortical activity [19]. This time delay between blood GCs and the appearance of metabolites in feces is species-specific [20]. Specifically, in deermice, [21] found a delay of 4 h before there were trap-induced effects on FCMs, but as they pointed out, the effects could have appeared sooner because they did not sample during a shorter interval (<4 h).

In this study, we had three objectives. Firstly, in the laboratory, we wanted to validate two different EIAs in measuring FCMs using physiological challenges (i.e., dexamethasone and ACTH injections). The immunoassays were a corticosterone EIA [12] and a 5α-pregnane-3β,11β,21-triol-20-one EIA (hereafter referred to as the group-specific EIA, [15]). Secondly, again in the laboratory, we wanted to use the diurnal rhythm in GC secretion to biologically validate both EIAs. Thirdly, in the field, we wanted to investigate temporal effects of trap confinement on FCMs using both EIAs to provide additional biological validation. 

## 2. Materials and Methods

### 2.1. Acquisition and Husbandry of Laboratory Deermice

We acquired 4 adult deermice (2 females: 2 males) from McMaster University, Ontario, Canada, and transported them to the animal holding facility at the University of Toronto—Scarborough, ON, Canada, in April 2016. These deermice were F1 generation offspring from wild deermice that were originally captured from Nebraska, USA [8]. All were ear-tagged, weighed, and sexed. All deermice were non-reproductive (males were non-scrotal, and females had non-perforate vaginas). They were individually housed in polypropylene cages (47 cm × 26 cm × 20 cm) that were equipped with a wire bottom and a glass water bottle with a stainless-steel nipple. All cages were mounted within a second same-sized cage that was equipped with a fine metal mesh. The wire bottom allowed feces and urine to fall through the bottom of the first cage. Though urine continued to pass through the fine mesh, the feces did not. This arrangement minimized urine contamination of feces and disturbance to the animals [22]. Male and female deermice were kept separate on two different but opposite racks within the same animal facility to limit exposure to odor from the opposite sex. We provided them with *ad libitum* water, rodent chow (LabDiet, St. Louis, MO, USA) and ample cotton bedding as nesting material. Deermice were housed under a 12:12 h dark-light cycle (lights on at 08:00 h) at room temperature (20 ± 5 °C). Ventilation fans were positioned in the wall at either end of the holding facility and operated in a push-pull method (the fan at one end pulled air out of the facility and the other pushed outside air into the facility). This method changed the air in the room 13 times/h. The direction of air flow was parallel to cage racks, which prevented cross contamination between cages that held males and females [23]. 

### 2.2. Fecal Sample Collection

We collected fecal samples by following Table 1 and discarded feces contaminated with urine. Forceps were disinfected between individuals during sample collection. If fecal pellets were in excess for an individual, we subsampled to get a representative pooled sample from all areas where the individual had defecated. We then stored samples at −20 °C until analyses. During the acclimation period, fecal samples were collected every 2 h (during the dark and light cycles) whereas during the challenge experiments, samples were generally collected every 2 h during the dark cycle and every 4 h during the light cycle (Table 1). This sampling change occurred due to personnel constraints.

### 2.3. Acclimation Period

Fecal samples were collected for ~92 h after the animals were transferred to our facility (Table 1). We assumed that samples collected on the last day of acclimation reflected baseline FCMs given that previous work with wild meadow voles (*Microtus pennsylvanicus*) found that FCMs were the lowest by the end of the third day of captivity [24]. 

### 2.4. Dexamethasone Suppression Challenge

To test whether EIAs could detect an expected decrease in FCM levels, we injected all deermice with 2.5 mg/kg dexamethasone sodium phosphate (Vétoquinol, Québec, Canada) diluted in sterile 0.9% saline intraperitoneally at ~20:00 h. In this way, each individual was used as its own control. Because dexamethasone doses have not been reported for deermice, we formulated this dose based on studies with other small rodents (e.g., [25,26,27]). We started to collect samples at 22:00 h for ~48 h although we were unable to analyze samples 12 h post injection (Table 1). No samples were collected at the time of injection. 

### 2.5. ACTH Stimulation Challenge

To test whether EIAs could detect an expected increase in FCM levels, we injected all deermice with 250 µg/ kg ACTH (Cortrosyn, Amphastar Pharmaceuticals, Inc., Rancho Cucamonga, CA, USA) mixed in 0.9% sterile saline solution intraperitoneally at ~20:00 h. Again, each individual was used as its own control and because ACTH doses have not been reported for deermice, we formulated this dose based on above cited studies. As above, we started to collect samples at 22:00 h for ~48 h although we were unable to analyze samples 12 h post injection (Table 1). No samples were collected at the time of injection. 

### 2.6. Field Validation

We carried out two field studies to assess the temporal effect of trap confinement on FCM levels. In both studies, deermice were captured in individual non-folding Sherman traps (H.B. Sherman Traps, Tallahassee, FL, USA) baited with oats and peanut butter, and provided with cotton bedding. In field study 1 (for group-specific EIA), apple slices were also provided. For field study 1, we trapped 20 adult deermice (7 males, 13 females) near Drummond, MT, USA, in June 2017. Only three were non-reproductive. Once trapped, we confined deermice in a trap for either 0–2, 4–6 or 8–10 h prior to processing. To do this, we set traps around dusk and checked them after 2 h. Trapped deermice were either processed for the 0–2 h treatment or left in the trap to be processed for the 4–6 h and 8–10 h treatments, where they spent an additional 4 h and 8 h in the trap, respectively. Deermice were removed from traps by “emptying” contents into a plastic bag. We then sexed, weighed, ear-tagged, and evaluated them for reproductive status. Age was determined based on mass (<14 g = juvenile, 14–17 g = subadult, >17 g = adult, [28]). Reproductive status was determined by the presence of scrotal testes in males, and presence of a perforate vagina, lactation, or pregnancy in females. 

In field study 2 (for corticosterone EIA), seven adult deermice (4 males, 3 females) were trapped near Charlo, MT, USA, in August 2017. Only two were non-reproductive. We checked traps after ~4 h of setup when we ear-tagged and collected feces from deermice. Afterwards, all deermice were returned to their respective clean traps where they spent the night until dawn (an additional ~7 h). At that time, they were sexed, weighed, evaluated for reproductive status, and sampled a second time for feces. Age and reproductive status were determined as above.

In both field studies, we collected feces from restrained animals and/or their trap and released individuals on site after processing. Feces contaminated with urine were not collected. We followed field protocols to avoid accidental hantavirus infection [29]. All fecal samples were stored at −80 °C until analysis. All procedures involving animal use were approved by the University of Toronto (protocol # 20011602) and/or by the University of Montana Institutional Animal Care and Use Committees (protocol #s 024-16ALDECS-042616, 027-16ALDECS-051016, 028-16ALDECS-051016). Field work was approved by Montana Fish, Wildlife and Parks (permit #2017-029-W). 

### 2.7. Processing of Feces and Extraction of FCMs

Laboratory and field study 1 fecal pellets were first oven-dried for 1 h at ~60 °C to heat-inactivate hantavirus (if present) and then lyophilized (Labconco Corp., Kansas City, MO, USA) for at least 15.5 h at Pennsylvania State University, State College, Pennsylvania, USA. Laboratory study fecal samples were not pulverized; they remained in pelleted form. However, in field study 1 we did pulverize, using a mortar and pestle, because there were fewer total samples (20 samples). To extract FCMs, we weighed 0.05 g (±0.005 g) of dried pellets/powder. Then, we added 1 mL of 80% methanol to each sample suspension, vortexed at 1500 RPM for 30 min, and centrifuged at 2500× *g* at 22 °C for 20 min [4,15]. Supernatants were decanted and frozen at −20 °C. The extraction of field study 2 samples was slightly different and performed at the University of Montana, Missoula, MT, USA. Fecal pellets (14 samples total) were heat-inactivated and oven-dried for 2 h at ~63 °C to ensure elimination of water, because a lyophilizer was unavailable. We pulverized dried pellets and weighed out 0.04 g (±0.005 g) of powder. The lower threshold weight was chosen because sample weights were generally lower in this field study. The rest of the extraction procedure remained unchanged. 

### 2.8. Immunoassay Methods

**Figure 1:**
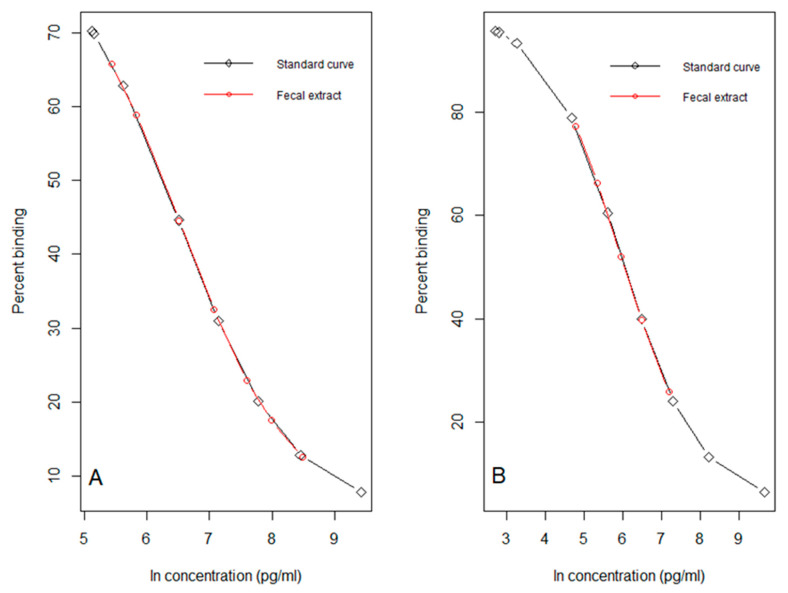
Parallelism curves for pooled fecal extract from deermice measured with (**A**) corticosterone enzyme immunoassay (EIA) and (**B**) group-specific EIA. Standard curves for each EIA are shown in black with each concentration as an open diamond. Parallelism is shown in red with serial dilutions of pooled fecal extract as open red circles.

For the analysis of FCMs two different EIAs were used. The immunoassays were a corticosterone EIA (commercial kit #K014-H1 or H5, provided by Arbor Assays, Ann Arbor, MI, USA) [12] and a 5α-pregnane-3β,11β,21-triol-20-one EIA (group-specific EIA, measuring FCMs with a 5α-3β,11β-diol configuration [15]). Details of the EIAs including cross-reactions are given by Arbor Assays and Touma et al. [15], respectively. All fecal extracts were diluted with EIA buffer prior to being analyzed. The dilution factor was determined by running pooled sample extract at different dilutions against the standard curve, to identify the one that resulted in ~50% binding for the corticosterone EIA (Figure 1A) and the group-specific EIA (Figure 1B). Through parallelism tests, we showed that serial dilutions of pooled extract tracked the EIA’s standard curve (Figure 1). These findings demonstrated that methanol residue in sample extracts did not interfere with assay performance. Consequently, sample extracts were diluted 1:10 for the corticosterone EIA, and 1:200 for the group-specific EIA. However, extracts from field study 2 analyzed with the corticosterone EIA had to be diluted 1:80 instead of 1:10 (change discussed later). 

We followed manufacturer’s instructions for the corticosterone EIA. However, when we analyzed fecal samples with this EIA for field study 2, we also used a wavelength of 650 nm (reference wavelength) in addition to 450 nm. We followed the protocol described by Touma et al. [15] for the group-specific EIA. All samples were assayed in duplicate. Intra-assay coefficients of variation (CVs) were calculated by averaging all sample CVs and inter-assay CVs were calculated by averaging CVs of low and high concentration controls for all plates (except for field study 2, we could only calculate average of low concentration controls). Intra-assay and inter-assay CVs for the corticosterone EIA were (*n* = 9) 5.7% and 12.7% (field study 2: 12.8%) and for the group-specific EIA (*n* = 6) 8.6% and 8.6%, respectively. 

### 2.9. Statistical Analyses

All data analyses were done in R [30] within RStudio [31]. We used linear mixed effect models (LMMs) from R packages “lme4” [32] and “lmerTest” [33] to test for diurnal patterns and how each treatment (dexamethasone suppression and ACTH stimulation) affected FCM levels of laboratory-bred deermice compared to baseline. We considered FCMs collected on the last day of acclimation as baseline FCMs. Because deermice were sampled repeatedly, individual identification was included as a random effect, where the model structure was sex + treatment × sampling time, except for when testing for diurnal patterns, which was sex + sampling time. *p*-values were calculated using Satterthwaite’s method [33]. We used the R package “emmeans” [34] to perform post hoc pairwise comparisons where *p*-values were adjusted accordingly. 

For field study 1, we used a one-way ANOVA with post hoc Tukey’s Honest Significant Differences to compare FCM levels across trap confinement treatments (i.e., 0–2, 4–6 and 8–10 h), sex and reproductive status, where the model structure was treatment + sex + reproductive status. For field study 2, we used a LMM to examine the difference between FCMs across two sampling times (i.e., 0–4 h vs. overnight), sex, and reproductive status, where the model structure was treatment + sex + reproductive status. Because each deermouse was sampled twice, individual identification was included as a random effect. 

FCM data were ln-transformed prior to all analyses to meet the assumptions of normality and homoscedasticity. Below, we present ln-transformed means with standard errors (ln ng/g of dry feces) where we considered results statistically significant at α = 0.05. However, in the figures we present non-transformed data to ease comparisons with other studies. 

## 3. Results

### 3.1. Acclimation

**Figure 2:**
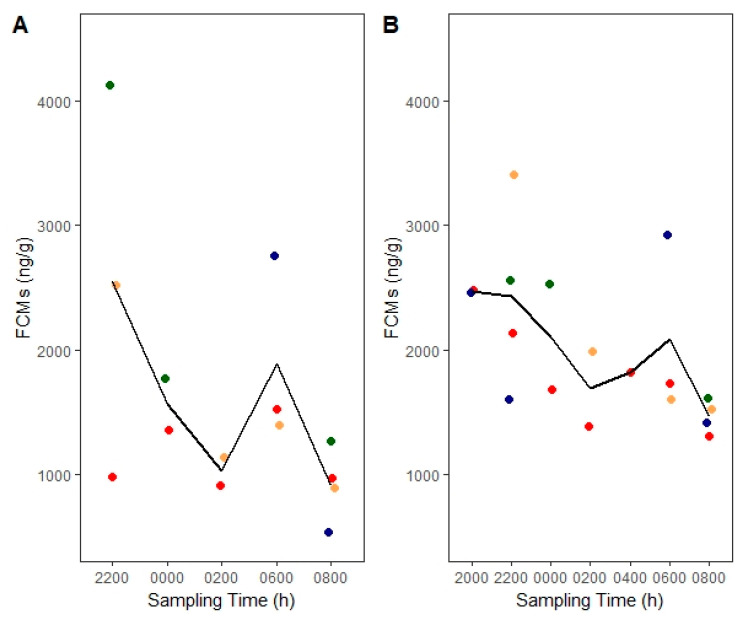
Changes in corticosterone baseline fecal cortisol/corticosterone metabolites (FCMs) from laboratory-bred deermice across the dark cycle measured with (**A**) corticosterone enzyme immunoassay (EIA) and (**B**) group-specific EIA. Lines connect means from each sampling time and circles indicate data points whereby an individual is denoted with a different color. Males are shown in blue and green colors, whereas females are shown in red and tan.

We did not find changes across sex (*p* = 0.16) or time (*p* = 0.10) with the corticosterone EIA (Figure 2A). Similarly, there were no changes across sex (*p* = 0.60) or time (*p* = 0.22) with the group-specific EIA (Figure 2B). However, it is noteworthy that the variability in FCMs appeared smaller towards the end of the dark cycle compared to the beginning. FCMs collected during the third day of acclimation were considered as baseline when we evaluated the effects of treatments (dexamethasone and ACTH).

### 3.2. Dexamethasone Suppression Test

**Figure 3:**
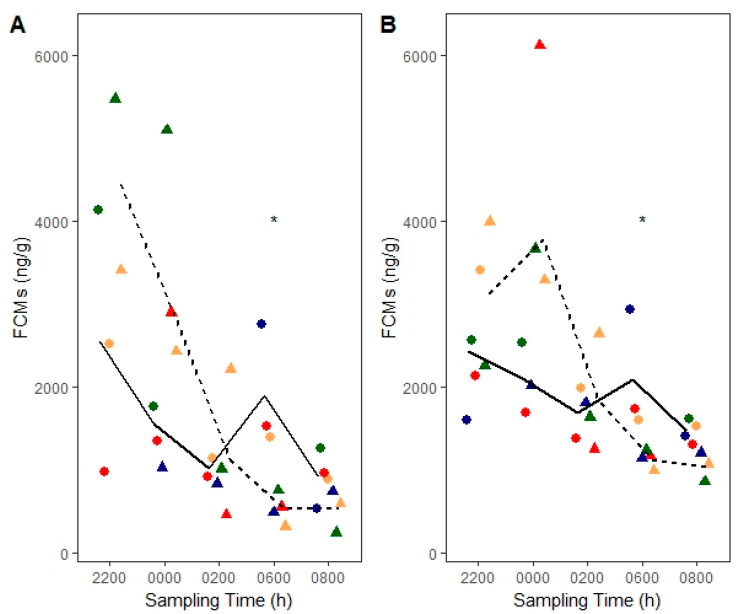
Corticosterone baseline fecal cortisol/corticosterone metabolites (FCMs) (solid lines and circles) versus post dexamethasone FCMs (dashed lines and triangles) in laboratory-bred deermice measured with (**A**) corticosterone enzyme immunoassay (EIA) and (**B**) group-specific EIA. Lines connect means from each sampling time. Circles and triangles indicate individual data points whereby an individual is denoted with a different color. Males are shown in blue and green colors, whereas females are shown in red and tan. Dexamethasone was administered at ~20:00 h (not shown). Asterisk (*) denotes significant differences at α = 0.05.

We found a sampling time by treatment effect for the corticosterone EIA (*F*_4, 18.31_ = 3.73, *p* = 0.02; Figure 3A). Deermice had lower FCM levels (*n* = 4, 6.23 ± 0.26 ln ng/g) ~10 h post injection than baseline (06:00 h, *n* = 3, 7.54 ± 0.30 ln ng/g, post hoc, *t*_18.32_ = −3.37, *p* = 0.003). We also found a sampling time by treatment effect for the group-specific EIA (*F*_4, 21_ = 3.39, *p* = 0.03; Figure 3B). Deermice had lower FCMs (*n* = 4, 7.03 ± 0.15 ln ng/g) than baseline ~10 h post injection (06:00 h, *n* = 3, 7.60 ± 0.17 ln ng/g, post hoc, *t*_19.46_ = −2.47, *p* = 0.02). Interestingly, deermice showed a marginal increase in FCMs (*n* = 4, 8.16 ± 0.15 ln ng/g) ~4 h post injection compared to baseline (*n* = 2, 7.63 ± 0.22 ln ng/g, *t*_20.42_ = 1.96, *p* = 0.06). We note that baseline FCM levels at 06:00 h represent a 2 h interval (04:00–06:00 h) whereas treatment FCM levels at the same time represent a 4 h interval (02:00–06:00 h; Table 1).

### 3.3. ACTH Stimulation Test

**Figure 4:**
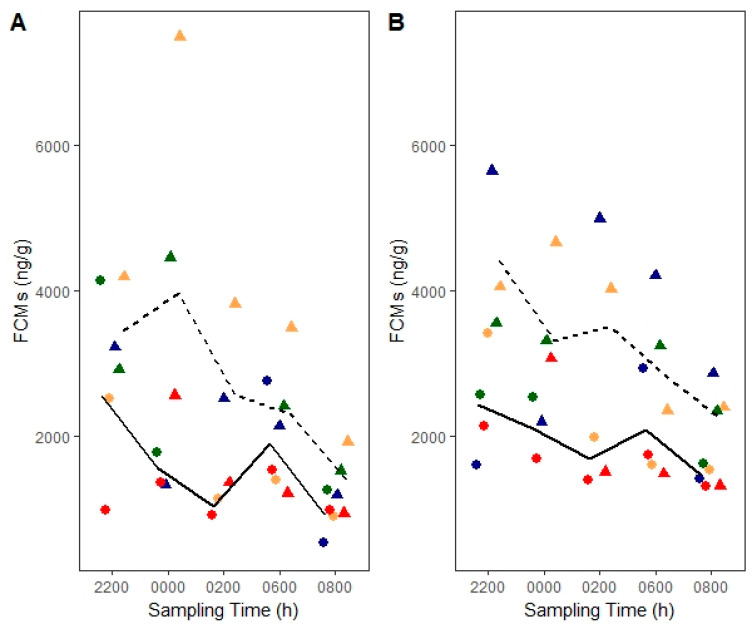
Corticosterone baseline fecal cortisol/corticosterone metabolites (FCMs) (solid lines and circles) versus post adrenocorticotropic hormone (ACTH) FCMs (dashed lines and triangles) in laboratory-bred deermice measured with (**A**) corticosterone enzyme immunoassay (EIA) and (**B**) group-specific EIA. Lines connect means from each sampling time. Circles and triangles indicate individual data points whereby an individual is denoted with a different color. Males are shown in blue and green colors, whereas females are shown in red and tan. ACTH was administered at ~20:00 h (not shown).

We found an effect of treatment for the corticosterone EIA (*F*_1, 19.22_ = 11.94, *p* = 0.003; Figure 4A) where deermice had consistently higher FCM levels (7.77 ± 0.19 ln ng/g) post injection than baseline (7.27 ± 0.20 ln ng/g, post hoc, *t*_19.17_ = 3.44, *p* = 0.003). We also found an effect of sampling time (*F*_4, 19.19_ = 5.42, *p* = 0.004) where deermice had higher FCM levels at 22:00 h (7.84 ± 0.23 ln ng/g; *t*_19.07_ = 4.06, *p* = 0.005), 00:00 h (7.74 ± 0.24 ln ng/g; *t*_19.17_ = 3.45, *p* = 0.02), and 06:00 h (7.62 ± 0.22 ln ng/g; *t*_19.05_ = 3.14, *p* = 0.04) compared to 08:00 h (6.99 ± 0.22 ln ng/g) regardless of treatment. Similarly, using the group-specific EIA, we found an effect of treatment (*F*_1, 20.25_ = 14.16, *p* = 0.001; Figure 4B) where deermice had higher (7.99 ± 0.12 ln ng/g) FCM levels consistently post injection than baseline (7.58 ± 0.12 ln ng/g, *t*_20.16_ = 3.75, *p* = 0.001; Figure 4B). Again, we also found an effect of sampling time (*F*_4, 20.28_ = 3.42, *p* = 0.03) where deermice had higher FCM levels at 22:00 h (8.02 ± 0.15 ln ng/g) compared to 08:00 h (7.48 ± 0.14 ln ng/g, *t*_20.06_ = 3.50, *p* = 0.02) regardless of treatment. We note that baseline FCM levels at 06:00 h represent a 2 h interval (04:00–06:00 h) whereas treatment FCM levels at the same time represent a 4 h interval (02:00–06:00 h; Table 1). 

### 3.4. Field Validation

**Figure 5:**
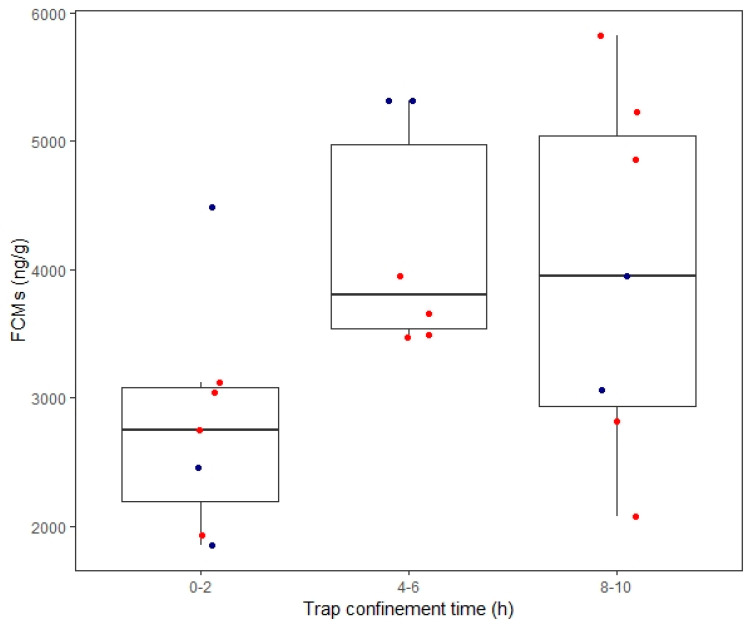
Fecal cortisol/corticosterone metabolites (FCMs) across different trap confinement times in free-ranging deermice. FCMs were measured with the group-specific EIA. Boxplots display the median (line), 25–75% interquartile range (boxes) and the full range (whiskers). In the 0–2 h group, there is a large outlier. Circles indicate individual data points whereby blue denotes males and red denotes females.

For field study 1 (group-specific EIA), we found that there was a marginal effect of confinement time on FCM levels (*F*_2,15_ = 3.38, *p* = 0.06), which we still elected to explore because of likely biological relevance. We found no effect of sex (*p* = 0.56) or reproductive status (*p* = 0.39). Deermice confined for 4–6 h had marginally higher FCM levels (*n* = 6, 4 females and 2 males, 8.33 ± 0.08 ln ng/g) compared to those confined for 0–2 h (*n* = 7, 4 females and 3 males, 7.90 ± 0.11 ln ng/g, *p* = 0.07). However, deermice confined for 0–2 h had no differences in their FCM levels compared to those of deermice confined for 8–10 h (*n* = 7, 5 females and 2 males, *p* = 0.16) or 4–6 h versus 8–10 h (*p* = 0.85; Figure 5). For field study 2 (corticosterone EIA), we found a significant effect of confinement time on FCM levels (*F*_1, 10_ = 23.21, *p* = 0.001). Deermice had lower FCMs (9.12 ± 0.32 ln ng/g) when confined for 0–4 h compared to after short-term restraint and overnight confinement (*n* = 7, 11.10 ± 0.32 ln ng/g, *t*_6_ = 4.82, *p* = 0.003; Table 2). Sex (*p* = 0.60) and reproductive status (*p* = 0.75) were not significant.

## 4. Discussion

We provided evidence that validates the use of both the corticosterone and the group-specific EIAs with FCMs in deermice. In the laboratory studies, both EIAs showed a similar decrease and increase in FCMs post dexamethasone and ACTH injections, respectively. Despite the group-specific EIA’s ability to detect particular corticosterone metabolites in feces, FCM values were comparable to the ones we detected with the corticosterone EIA. Field study 1 (group-specific EIA) showed that deermice had marginally higher FCM levels when confined for 4–6 h versus 0–2 h. Field study 2 (corticosterone EIA) more strongly echoed these results where deermice had higher FCMs after short-term restraint and confinement more than 0–4 h. Although we did not verify whether the stressors we used increased blood corticosterone, we do not think this affects our conclusions because of two main reasons. Firstly, the stressors we used have been known to influence blood GCs in other species (e.g., [18,35]), and secondly, many other validation studies for FCMs were successful without performing this type of verification (e.g., [15,36,37,38]). Although comparing between studies with different extraction and assay protocols is difficult, both EIAs we used consistently detected higher values compared to the corticosterone RIA used in previous deermouse studies (e.g., [10,11]). 

### 4.1. Diurnal Rhythm and Sex Effects

We detected no effects of diurnal rhythm or sex on FCMs with either EIA when using only data from the third day in captivity. However, the variability in FCM data was smaller towards the end of the dark cycle compared to the beginning, suggesting that if we had FCM data from each deermouse for each time point, a significant change over time may have manifested. Regardless, we did find higher FCMs at 22:00 h compared to 08:00 h when using pooled data from the ACTH challenge for both EIAs. In fact, FCMs were also higher at 00:00 h and 06:00 h compared to 08:00 h for the corticosterone EIA. This finding from pooled data is most likely because, although treatment FCMs were relatively higher than baseline, they still showed a declining trend, similar to baseline FCMs, across the dark cycle. Previous studies with small mammals found either a presence or absence of a diurnal rhythm in FCMs (e.g., [24,25]). However, when a diurnal rhythm is found, FCM levels will typically rise before the period of highest activity and start to decrease closer to the period of inactivity, which is similar to what we found [25,39]. This reflects the dynamics of blood GCs before they appear in feces, which is governed by a species-specific time delay [4]. This delay can range from 4 h in small mammals (e.g., house mice, [15]) to ~24 h in larger mammals [20]. Similarly, [40] found no effect of sex on FCM levels in deermice, although [25] detected sex differences in house mice where females had higher FCM levels. Due to low sample size of females and males, there may have been an effect of sex on FCMs, which we were unable to detect.

### 4.2. Suppression of Adrenocortical Activity

We found that FCM levels decreased significantly ~10 h post dexamethasone injection with both EIAs. FCMs decreased on average by ~73% and ~43% for the corticosterone and group-specific EIAs, respectively. This however, could have happened sooner (i.e., ~8 h post injection) since we lacked FCM data at 04:00 h. Nevertheless, other rodent studies found FCM levels decreased 8-10 h post dexamethasone in house mice [25] and 10–12 h in Norway rats (*Rattus norvegicus*) [41], although injections were given during the light cycle. However, the percentage decreases we observed were lower than in Norway rats (~86%; [41]) but higher than in Columbian ground squirrels (*Urocitellus columbianus*) (~33%; [36]), both of which used the same group-specific EIA. This could suggest that a higher dexamethasone dose could be used to more strongly suppress FCMs in deermice. It is noteworthy that the group-specific EIA did detect a marginal increase in FCM levels ~4 h post dexamethasone injection (~69% average increase), most likely due to restraint/injection stress. Even if a higher dose may have resulted in a larger effect size, both EIAs tracked the expected suppression in FCMs post dexamethasone.

### 4.3. Stimulation of Adrenocortical Activity

FCM levels increased ~2 h post ACTH injection and remained elevated when using both EIAs. In particular, FCMs on average increased by ~65% and ~50% with the corticosterone and group-specific EIAs, respectively. Given that other rodent studies found longer time delays than 2 h post ACTH injection, such as 5–7 h in Egyptian spiny mice (*Acomys cahirinus*) [42] and 6–8 h in bank voles (*Myodes glareolus*) [43], this finding was unexpected. However, in these studies the ACTH injections were given during the light cycle in rodents that are mostly nocturnal, which could have affected time delays [15]. Regardless, [44] did find that brown lemmings (*Lemmus trimucronatus*) reached their half maxima FCM values within 2 h of capture, anesthesia, and transportation in the field. Similarly, [26] found that fecal radioactivity appeared as early as 2 h in a radiometabolism study of California mice (*Peromyscus californicus*). Nonetheless, in our study, it is still possible that capture and restraint for injection significantly decreased gut passage time [4]. The percentage increases we found are much lower than previous mammal studies. For example, [36] found ~255% increase post ACTH in Columbian ground squirrels using the group-specific EIA. However, [24] found ~56% increase in meadow voles using the group-specific EIA. Therefore, the ACTH dose we used may not have been high enough to reach a stronger effect. Regardless, the modest yet significant increase in FCM levels post ACTH injection provides validation evidence for both EIAs. 

### 4.4. Trap-Induced Effects on FCMs

Trap confinement for 4–6 h marginally increased FCM levels in free-ranging deermice, compared to confinement for 0–2 h but not compared to 8–10 h (field study 1). Similarly, deermice confined for 0–4 h had lower FCM levels compared to additional confinement of ~7 h and after short-term restraint (field study 2). Although we cannot easily tease apart effects from restraint and trap confinement time in field study 2, the findings still provide biological validation. Because FCM levels tend to decrease shortly into the active phase [25,39], the elevations we observed after 4 h would most likely have been due to trap-induced stress. The lack of difference between 0–2 h and 8–10 h could stem from how the stressor of trap confinement remained consistent over time so that FCMs eventually returned to baseline. Alternatively, it could be that the natural decline of FCMs overnight conflicted with the increase in FCMs from trap-induced stress, thereby leading to a lower average FCMs and a larger variability in the data for the 8–10 h group (Figure 5). Similarly, [21] found no differences in FCM levels between deermice in traps for 4–8 h versus overnight, although FCM levels did continue to increase with more trap confinement in another deermouse population. Based on field study 1 findings, the lag time between corticosterone in the blood to excretion in the feces may be ~4 h during the period of highest activity (i.e., dark cycle). This is similar to what has been reported in another deermouse study that used a corticosterone RIA [21]. Although sex and reproductive status can influence stress physiology [4], we did not find any effects on FCMs from sex or reproductive status. However, this may have been due to low sample sizes, and not a limitation of the EIAs. Nevertheless, our findings suggest that trap-induced stress may affect FCM levels even within 4 h of confinement so that earlier fecal collection may better capture baseline adrenocortical activity and unmask individual heterogeneity. 

### 4.5. Drying Effects on FCMs

Although samples oven-dried for 1 h and then lyophilized were diluted 1:10 for the corticosterone EIA, samples oven-dried for 2 h with no lyophilization had to be diluted 1:80 instead. This increase in the dilution factor could be the result of additional drying time where further alteration of FCMs can affect actual FCM levels and influence antibody binding. Similar heat effects on FCM levels were reported by [45] where autoclaving ungulate feces artificially increased FCM levels. However, an alternative reason could be the origin of the samples because those that were diluted more came from free-ranging deermice whereas those that were diluted less came from laboratory deermice on rodent chow diet. Because diet can affect FCM levels, the diet of free-ranging deermice may have led to artificially higher FCM levels [46]. However, because the group-specific EIA did not detect differences between laboratory and wild deermice (i.e., same dilution factor), it is most likely that an additional hour of oven-drying induced structural changes to FCMs that were detected by the corticosterone EIA antibody, thereby increasing FCM levels (e.g., [47]). Therefore, the drying protocol needs to remain consistent throughout a study, (e.g., for multiple samples from one individual) if valid FCM comparisons are to be made.

## 5. Conclusions

We analytically, physiologically, and biologically validated two EIAs for measuring FCMs in deermice. Although we used identical sample processing and extraction methods for laboratory samples, this was not the case with field samples so direct comparisons should be made with caution. Nevertheless, both field studies demonstrated similar temporal patterns, so they provided biological validation for the two EIAs. Although we acknowledge low sample sizes, our study is the first to provide validation evidence for EIAs that can quantify FCMs in deermice.

## Figures and Tables

**Table 1:** Timeline of treatments and samples used in statistical analyses for evaluation of fecal corticosterone metabolites in deermice.

Date	Treatment ^1^	Immunoassay	Sample Collection ^2^ Schedule (h Post Treatment)
May 2–3	Acclimation	Corticosterone EIA	70, 72, 74, 76, 80, 82
		Group-specific EIA	70, 72, 74, 76, 78, 80, 82
May 3–4	Adrenal suppression	Corticosterone EIA	2, 4, 6, 10, 12
		Group-specific EIA	2, 4, 6, 10, 12
May 5–6	Adrenal stimulation	Corticosterone EIA	2, 4, 6, 10, 12
		Group-specific EIA	2, 4, 6, 10, 12

^1^ Injections administered at ~20:00 h, ^2^ Acclimation lasted for ~92 h whereas adrenal treatments lasted for ~48 h (see text for details).

**Table 2:** Summary of demographic factors and fecal cortisol/corticosterone metabolite (FCM) values from free-ranging deermice captured in MT, USA in August 2017 for field study 2.

Sex	Reproductive ^1^	FCMs at 0–4 h (ng/g)	FCMs Overnight (ng/g)	FCM Difference ^2^
Male	No	21,034	49,709	28,676
Male	No	5407	76,071	70,663
Male	Yes	11,224	185,928	174,704
Male	Yes	4649	91,514	86,865
Female	Yes	37,172	37,776	604
Female	Yes	3611	41,678	38,067
Female	Yes	9704	82,299	72,595

^1^ Reproductive status was determined via scrotal testes in males and presence of a perforate vagina, lactation, or pregnancy in females, ^2^ FCM difference was calculated by subtracting FCMs at 0–4 h from FCMs overnight.
